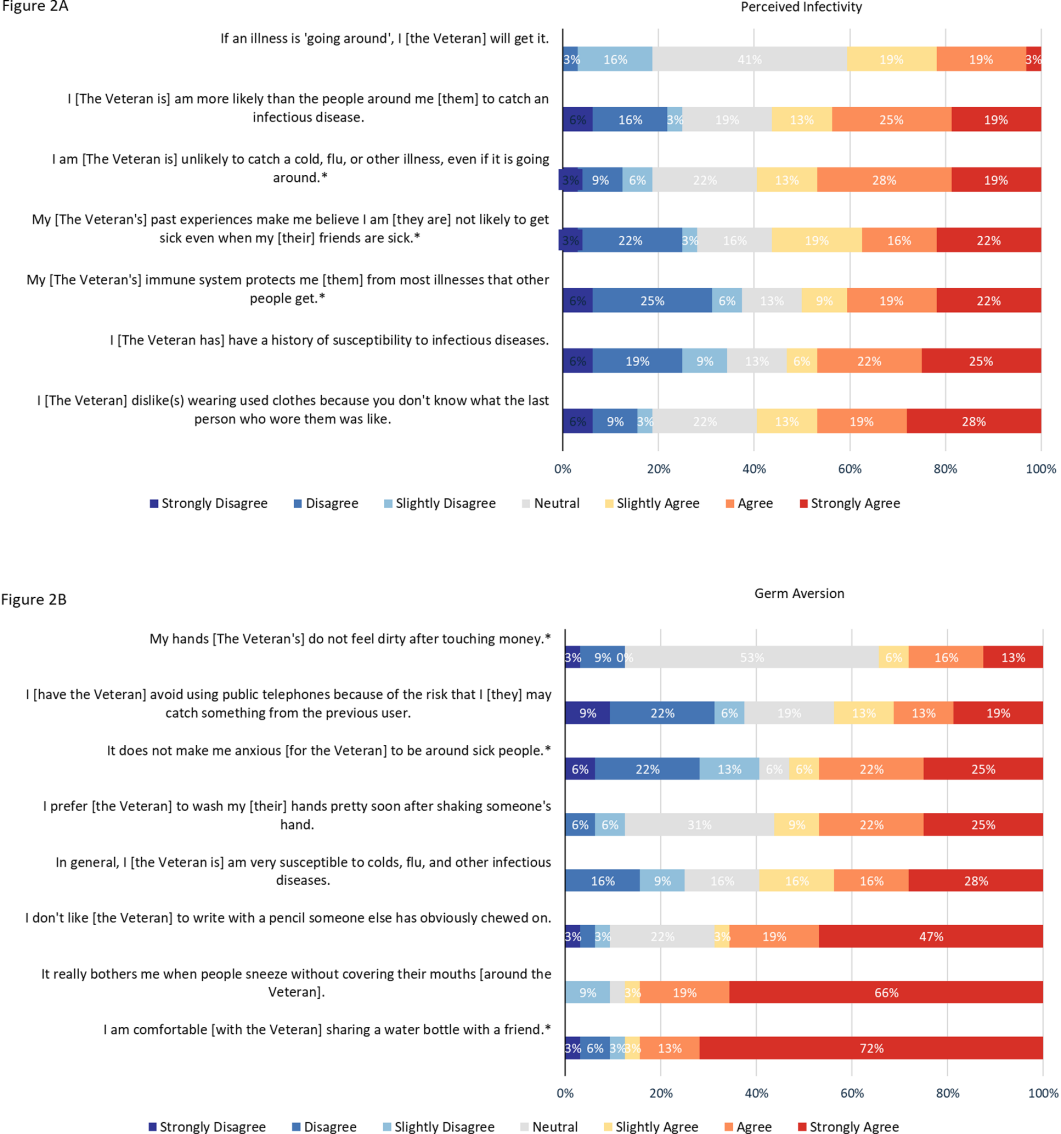# 369 Impact of Algorithm-Guided Management of Clostridioides difficile Infection in Hospitalized Patients

**DOI:** 10.1017/ash.2026.10705

**Published:** 2026-06-23

**Authors:** Shaye Kerper, Robin Jump, Mary Connolly, Alison O’Donnell

**Affiliations:** 1 University of Pittsburgh; 2 VA Pittsburgh Healthcare System; 3 UPMC

## Abstract

**Background:** Residents living in post-acute and long-term care settings (PALTC) settings are particularly vulnerable to severe infections caused by common respiratory viruses, including COVID-19, influenza, and respiratory syncytial virus (RSV). The Perceived Vulnerability to Disease (PVD) scale has been used to understand general public perceptions of vulnerability to infectious diseases. We adapted the PVD for administration to PALTC residents and their care partners. Here, we describe the perceptions of infectious disease risk among Veterans and their care partners at a large Veterans Affairs (VA) PALTC setting, termed Community Living Centers (CLCs). **Methods:** From November 2024 to July 2025, we surveyed residents at a 152-bed CLC. Inclusion criteria were CLC residents aged > **Results:** Out of 81 eligible residents and LARs, 40% (N=32; 6 residents and 26 LARs) completed the survey. Regarding previous infections, 27 (84%), 9 (28%), and 0 (0%) respondents indicated that Veterans had tested positive for COVID-19, influenza, or RSV, respectively, during their CLC stay. No more than 9 (28%) perceived a moderate to high risk of infection with any of the pathogens during the next month (Figure 1). At least 12 (38%) of respondents agreed or strongly agreed with 6 of the 7 items for the Perceived Infectivity subscale of the PVD; at least 15 agreed or strongly agreed with 6 of 8 items for Germ Aversion (Figure 2). **Conclusions:** Participant responses to the Perceived Infectability and especially Germ Aversion indicated concerns with personal susceptibility to illness and general contamination. The perceived risk specifically to COVID-19, influenza, and RSV was comparatively lower, despite most Veterans having viral respiratory infections during their CLC stay. Although based on a small sample, our findings provide valuable insight about resident and LAR perspectives that can help inform and support infection prevention efforts in PALTC settings.

**Figure:**
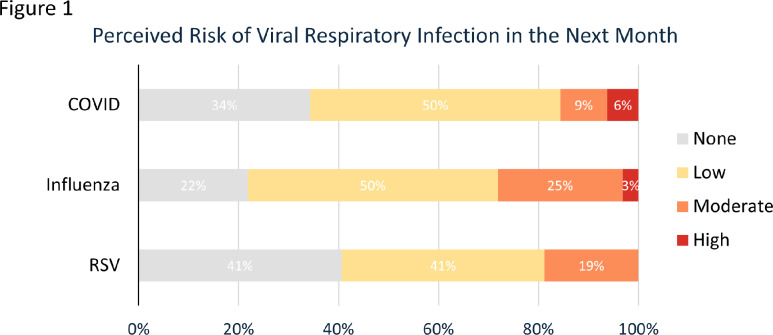

**Figure:**